# When does metacognition evolve in the opt-out paradigm?

**DOI:** 10.1007/s10071-024-01910-5

**Published:** 2024-10-22

**Authors:** Robin Watson

**Affiliations:** 1 School of Human Evolution and Social Change, Arizona State University, 900 South Cady Mall, Tempe, AZ 85287 USA; 2 Institute of Human Origins, Arizona State University, 777 E University Drive, Tempe, AZ 85287 USA; 3 Present Address: School of Psychology, University of Lincoln, Sarah Swift Building, Brayford Wharf East, Lincoln, LN5 7AT UK

**Keywords:** Evolution of metacognition, Evolutionary model, Opt-out paradigm, Theory of mind, Uncertainty monitoring

## Abstract

Metacognition (awareness of one’s own knowledge) is taken for granted in humans, but its evolution in non-human animals is not well understood. While there is experimental evidence of seemingly metacognitive judgements across species, studies rarely focus on why metacognition may have evolved. To address this, I present an evolutionary model of the opt-out paradigm, a common experiment used to assess animal’s metacognition. Individuals are repeatedly presented with a task or problem and must decide between opting-out and receiving a fixed payoff or opting-in and receiving a larger reward if they successfully solve the task. Two evolving traits – bias and metacognition – jointly determine whether individuals opt-in. The task’s reward, the mean probability of success and the variability in success across trials, and the cost of metacognition were varied. Results identify two scenarios where metacognition evolves: (1) environments where success variability is high; and (2) environments where mean success is low, but rewards are high. Overall, the results support predictions implicating uncertainty in the evolution of metacognition but suggest metacognition may also evolve in conditions where metacognition can be used to identify cases where an otherwise inaccessible high payoff is easy to acquire.

## Introduction

Metacognition broadly refers to a collection of processes through which individuals gain awareness of their own knowledge or cognition and the ability to use this knowledge adaptively (Schwartz et al. 2023; Smith et al. 2016; Templer 2022), for example by choosing to avoid a difficult task they know they have little chance of succeeding at. While metacognition is taken for granted in humans, the prevalence of metacognition within other species is unclear, though the body of indicative evidence in various species is growing (Templer 2022).

Studying metacognition in non-human animals is done through experiments, which typically involve recalling the correct stimulus or the location of food items after a delay. Information-seeking paradigms investigate whether individuals seek additional information in contexts where they are more likely to be uncertain (Crystal and Foote 2011; Iwasaki et al. 2018; Watanabe and Clayton 2016). Wagering paradigms offer individuals the opportunity to “bet” on their response, where using metacognition would predict more frequently betting on easier trials (Kornell et al. 2007; Middlebrooks and Sommer 2012; Nakamura et al. 2011). However, the most common approach is the opt-out paradigm, where individuals can choose to decline a difficult (but rewarding) task and instead attempt an easier less rewarding task or receive a fixed reward (Schwartz et al. 2023). Metacognition is inferred if individuals selectively opt-out on difficult trials and perform worse on trials without the option to opt-out (Crystal and Foote 2011; Schwartz et al. 2023). Through the opt-out paradigm, indications of metacognition have been observed in species such as dolphins (Smith et al. 1995), rats (Templer 2019), rhesus monkeys (Brown et al. 2019), and even honeybees (Perry and Barron 2013).

Despite the growing body of indicative evidence of metacognition in animals, it remains unclear under what conditions metacognition would be expected to evolve. Many studies focused on establishing the evidence of metacognition give little attention to why such abilities may have evolved (Schwartz 2019). To date, there have been no formal phylogenetic analyses (Schwartz et al. 2023), partly because many classes have never been studied (Lage et al. 2022; Templer 2022). Consequently, almost nothing is known about metacognition in many groups (Templer 2022). Additionally, aside from some specific examples such as Arctic foxes (Eaton et al. 2020), capuchins (Smith et al. 2018) and pigeons (Sutton and Shettleworth 2008; but see, Iwasaki et al. 2018), there are few empirical examples of a failure to demonstrate metacognition (Schwartz 2019).

Many speculative explanations of the evolution of metacognition highlight the importance of environmental uncertainty (Proust 2006; Smith et al. 2012). A general prediction is that species with predictable food sources and that interact with fewer conspecifics are less likely to evolve metacognition (Schwartz et al. 2023). Relatedly, competition for clumped food sources is suggested to have driven the evolution of social cognition in great apes (Tomasello 2023). However, in addition to the empirical gaps discussed above, there is a lack of formal evolutionary theory within which these hypotheses may be tested.

Evolutionary models are useful for addressing empirical discrepancies or providing novel testable predictions. For example, the positive relationship between lifespan and brain size (a proxy for increased cognitive ability) is strong in mammals (González-Lagos et al. 2010; Isler and van Schaik 2009), but weaker for other classes (Minias and Podlaszczuk 2017; Stark 2022). One model found that increased learning speed evolved when lifespan was long enough for individuals to learn to acquire a valuable trait but not so long that the trait could be acquired easily (Liedtke and Fromhage 2019). This highlighted a condition under which increased cognitive abilities could evolve despite a short lifespan. Here I present an evolutionary model with the principal goal of generating predictions regarding when and under what conditions metacognition would be expected to evolve in the opt-out paradigm. I vary: (1) the cost of metacognition; (2) the mean and (3) variation in task success probability; and (4) the reward associated with task success. I focus on the opt-out paradigm because it is the most used experimental method to study metacognition. Additionally, a recent review favoured the opt-out paradigm over the information-seeking paradigm for assessing individuals’ metacognition (Schwartz et al. 2023).

## Methods

I employ an evolutionary agent-based model using R (see Acerbi et al. 2020 and Smaldino 2023 for practical guides). In such models, a starting population of individuals are assigned allele values that influence their actions within a simulated environment. Across generations individuals are selected to reproduce according to their relative payoffs. This means allele values that result in higher payoffs are increasingly represented in subsequent generations. In addition, a small mutation rate ensures that all possible allele values could conceivably evolve.

I consider an environment where individuals repeatedly face the decision between opting-in/out of attempting a task across 100 timesteps. If they opt-in and are successful, they receive a large reward (*R*), but do not receive the reward if they are unsuccessful. Whereas if they opt-out, they receive a guaranteed payoff of 1. This closely emulates the design of experiments employing the opt-out paradigm (Schwartz et al. 2023).

At each timestep, the true probability of succeeding at the task, $\:{P}_{t}$, is sampled from a beta distribution, with mean *µ* and precision or “sample size” $\:\varphi\:$. To specify the shape of the distribution, *µ* and $\:\varphi\:$ are transformed to shape parameters (α, β). Here *µ* controls the average difficulty of the task and $\:\varphi\:$ controls the variability in the difficulty of the task. This could reflect the difference between an easy to acquire consistently available food item and an inconsistent difficult to acquire food item. Both *µ* and $\:\varphi\:$ remain constant throughout an individual’s lifetime.

$$\begin{array}{l}\alpha  = \mu {\rm{\Phi }}\\{\rm{\beta }} = \left( {1 - {\rm{\mu }}} \right){\rm{\Phi }}\\{{\rm{P}}_{\rm{t}}} \sim {\rm{B}}\left( {\alpha ,{\rm{\beta }}} \right){\rm{}}\end{array}$$

Individuals possess two evolving traits – bias (*B*) and metacognition (*M*). *B* can take any value between − 1 and 1 and reflects an individual’s innate predisposition to opt-in/out of the task. *M* takes any value between 0 and 1 and determines how accurately individuals’ can assess their success probability. Through the process explained below, these traits jointly determine whether an individual opts-in, where metacognition gives individuals the flexibility to opt-in/out depending on their chance of success. The cost of metacognition is scaled according to a cost parameter ($\:c$). This reflects the metabolic costs/caloric requirements of sustaining the neuronal material required for metacognition.

### Model steps

Steps 1–4 are repeated 100 times during an individual’s lifetime, meaning they make 100 opt-in/out decisions. This number was chosen because it was long enough to enable selection to dominate over drift without increasing the running time of the simulation unnecessarily.

1. **Assess task success probability** – Individuals first judge their expected probability of success if they were to opt-in to the task ($\:{P}_{j})$ by sampling from a normal distribution with a mean equal to the true success probability for that timestep $\:{(P}_{t})$. The standard deviation of this distribution is lowered according to their metacognition (1 – *M*).
  $$\:{\text{P}}_{\text{j}}\:\sim\:N\left({\text{P}}_{\text{t}},\:1-M\right)$$
  Their final expected probability of success ($\:{P}_{e})$ is determined by adding their bias (*B*) value to $\:{P}_{j}$. This means individuals could still be strongly inclined towards opting-in/out, regardless of their metacognition. Where $\:{P}_{e}$ extends below 0 or above 1, values are truncated.
  $$\:\:{\text{P}}_{\text{e}}={\text{P}}_{\text{j}}+B$$
2. **Calculate the expected benefit of opting-in** – The expected payoff ($\:E\left[b\right]$) of opting-in is summarised as:
  $$\:E\left[b\right]={P}_{e}*R$$
  where *R* is the reward received from succeeding at the task.
3. **Determine whether to opt-in** – Individuals opt-in if $\:E\left[b\right]>1$ but opt-out if $\:E\left[b\right]<1$ (the payoff from opting-out is 1). In the case that $\:E\left[b\right]=\:1$, individuals opt-in or opt-out randomly.
4. **Receive the payoff for that timestep** – If the individual opted-in, they succeed with probability $\:{P}_{t}$ and receive the reward (*R*), otherwise they receive no payoff. If they opted-out, they receive a fixed payoff of 1.
  After 100 timesteps, individuals proceed once through the following two steps.
5. **Determine their lifetime fitness** – Lifetime fitness $\:(\omega\:$) is the sum of their payoffs, minus the cost from their investment in metacognition.
  $$\:\omega\:=\:{\Sigma\:}\text{P}-\text{c}\text{M}$$
6. **Reproduction** – Individuals reproduce asexually in proportion to their fitness. Mutation is applied to *B* and *M*, whereby a value drawn from a normal distribution with mean 0 and standard deviation 0.01 are added to the trait values during reproduction. Values are truncated where they extend beyond the permitted range for each trait.

Four parameters were systematically varied (Table 1): mean task success probability (*µ)*, variation of task success probability ($\:\varphi\:$), metacognition cost (*c*), and the opt-in reward (*R*). The model was repeated 36 times for every combination of the parameter values. Each model ran for at least 250 generations but was stopped at equilibrium when bias and metacognition changed less than 0.02 (two mutation standard deviations) compared to 10 generations ago.

**Table 1:** Model parameters, how they are determined in the model, and the range of values they can take

Parameter	Type	Range
Mean task success probability (*µ*)	Varied	0.25, 0.5, 0.75
Variation task success probability ($\:\varphi\:$)	Varied	1, 2, 5, 10, 20
Cost of metacognition ($\:c$)	Varied	5, 10, 15, 20
Opt-in reward (*R*)	Varied	1.5, 2, 3, 4
Bias (*B*)	Evolving	-1–1
Metacognition (*M*)	Evolving	0–1

## Results

**Fig. 1:**
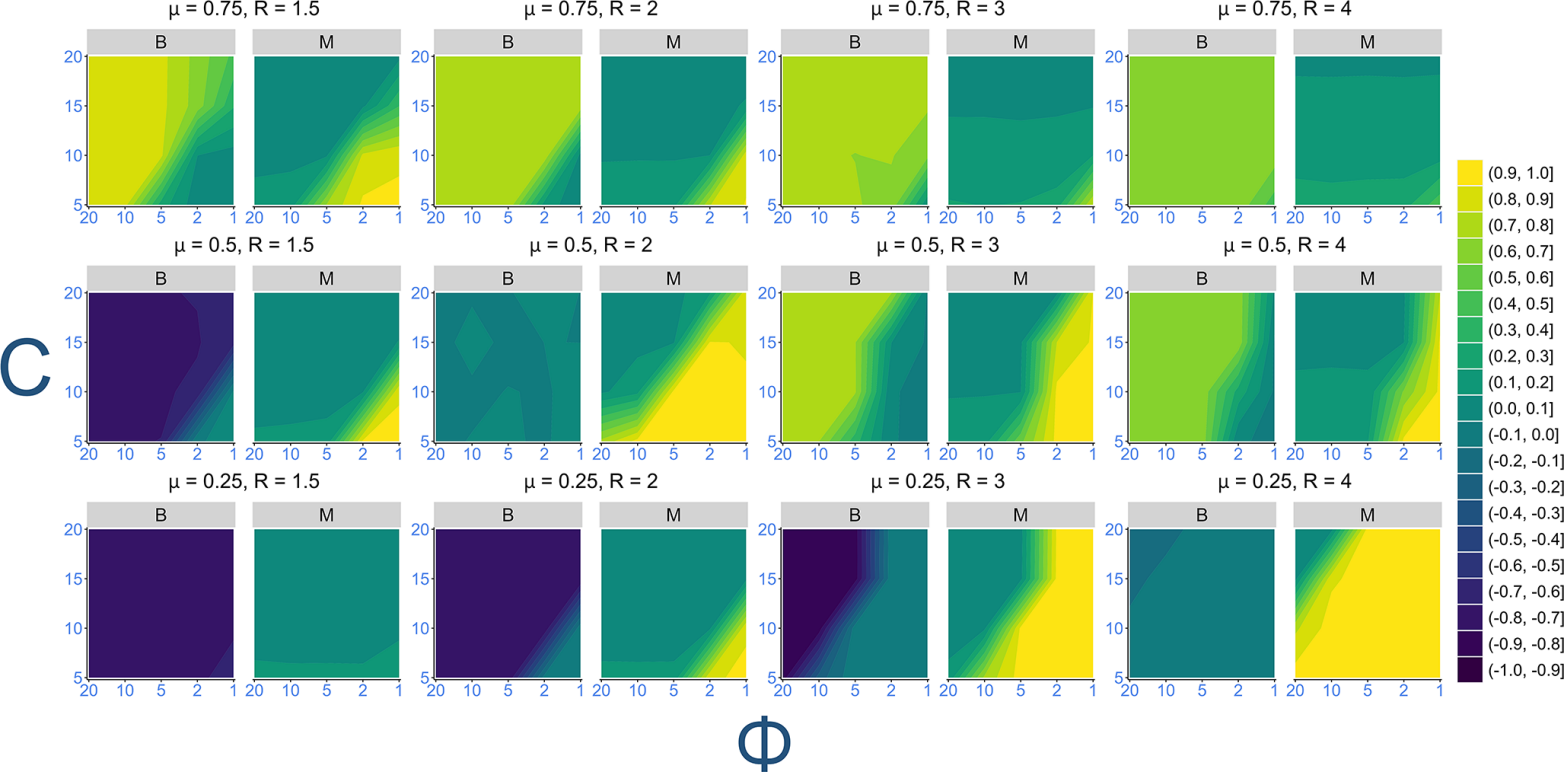
Contour plot showing mean bias and metacognition values at equilibrium. Grid rows vary *R* and grid columns vary $\:\mu\:$. *C* (y-axis) and $\:\varphi\:$ (x-axis) are varied within each plot. Lighter colours indicate higher values and darker colours indicate lower values. Row numbering referenced in the main text counts from the bottom row ($\:\mu\:$ = 0.25)

Mean trait values at equilibrium are shown in Fig. 1. Generally, metacognition evolves when task variability is high ($\:\varphi\:$ close to 1) alongside a neutral bias (*B* around 0). Otherwise, a positive bias (*B* > 0) evolves when opting-in is generally profitable but a negative bias (*B* < 0) when opting-in is generally unprofitable. In intermediate cases, whether metacognition evolves depends on the values of µ, *R* and *c*. The coevolution between bias and metacognition across different parameters is summarised below.

*High metacognition and neutral bias* evolve when task success probability and the opt-in reward are moderate (µ = 0.5, *R* = 2; row 2 columns 2). Increasing or decreasing *R* favours a reduction in metacognition and either an increase or decrease in bias respectively. This reflects cases where *R* is sufficiently high on average or too low overall that investing in metacognition is not worthwhile compared to uncritically opting-in/out. Metacognition is still favoured when variability is high ($\:\varphi\:$ close to 1) but under fewer conditions as *R* increases.

*High metacognition and* neutral bias evolve more strongly when the task is difficult but highly rewarding (µ = 0.25, *R* > 2; row 1, columns 3 and 4). When *R* is at its maximum, metacognition evolves strongly for almost all parameter values, being the only case where maximally costly metacognition consistently evolves. Here, because the task is difficult, it cannot be exploited by bias, meaning metacognition is required to spot cases where the task is unusually easy.

*Low metacognition and positive/negative bias* evolve when task variability is low ($\:\varphi\:$ close to 20). A consistently easy task (µ = 0.75; row 3) favours a positive bias, while a moderate or difficult task (µ = 0.5 or 0.25) favours a negative bias. In both cases, because the task success probability is very consistent, bias alone can handle the decision to opt-in/out. Note that for easy tasks, bias evolves to be more negative as *R* increases. This is because individual’s expected benefit is calculated based on *R*, meaning a more negative bias is needed to ensure individuals continue to opt-out even as the potential reward from succeeding increases.

*Low metacognition and neutral bias* evolve rarely, only when the expected benefits from opting-in/out are roughly equal, which occurs when variability is low (row 1, column 4; row 2, column 2).

**Fig. 2:**
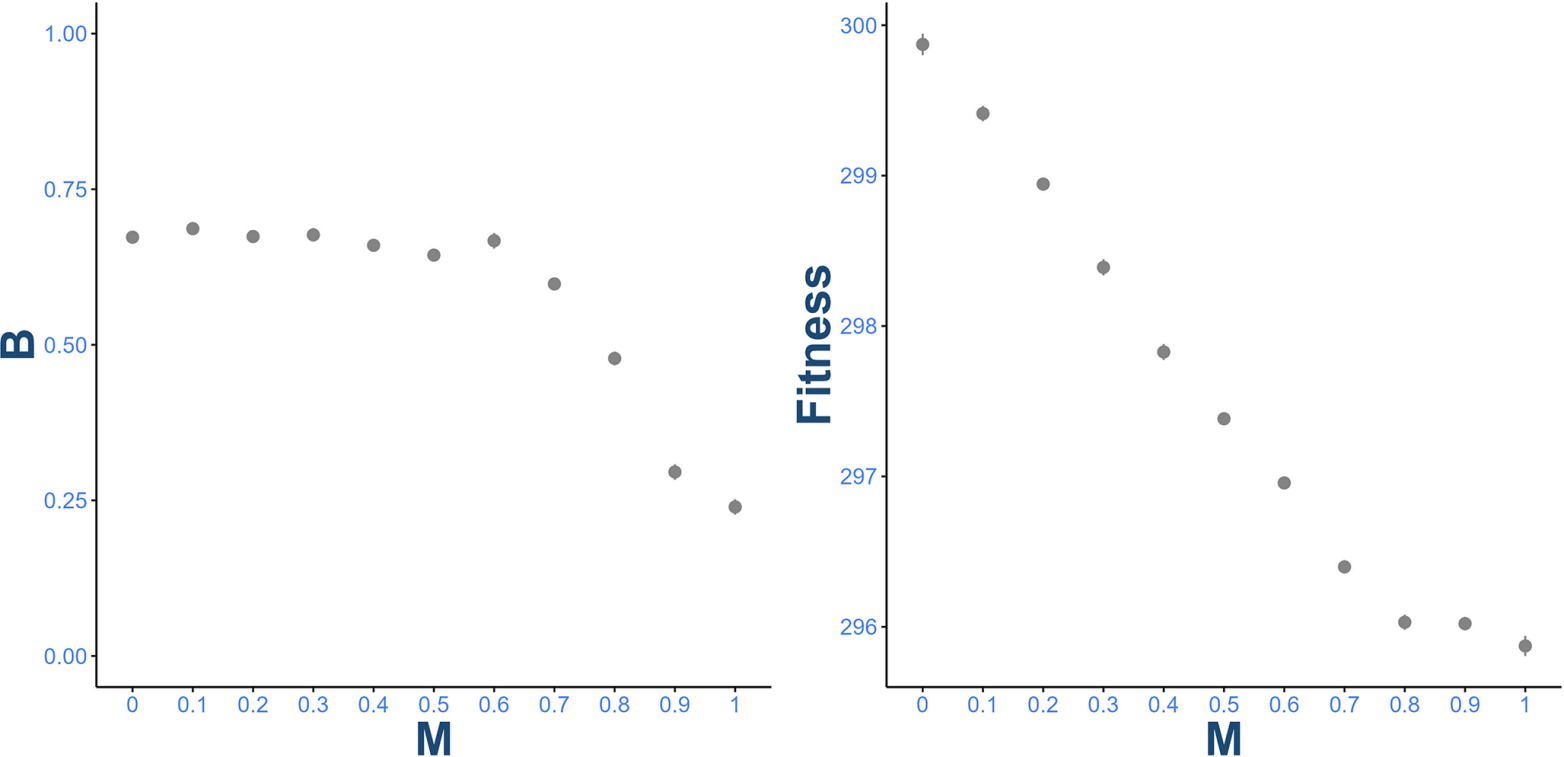
**Left**: mean and standard error equilibrium values for bias. **Right**: mean fitness for fixed levels of metacognition. Other parameters were fixed at: *c* = 5, $\:\varphi\:$ = 2, $\:\mu\:$ = 0.75, *R* = 4. Values are computed from 12 model repeats

Finally, and surprisingly, the combination of *moderate metacognition and positive bias* appears to evolve when the task is easy and rewarding ($\:\mu\:$ = 0.75, *R* > 1.5: row 3, columns 2, 3 and 4), particularly when metacognition is cheap ($\:c$ < 15). This is surprising, because bias overrides the estimate of success probability made through metacognition. To investigate this, further simulations were conducted where metacognition was fixed at equally distanced values between 0 and 1, but bias was allowed to freely evolve. While bias evolves to be less positive as metacognition increases, mean payoffs remain roughly the same in each case (Fig. 2). Therefore, selection on metacognition was weak under these conditions – evolving a higher bias and no metacognition or a lower bias and high metacognition offered equal payoffs.

## Discussion

Using an evolutionary model, I explored the conditions under which metacognition would be expected to evolve in the opt-out paradigm, a commonly used experimental method to study metacognition (Schwartz et al. 2023). In such experiments, individuals must choose between opting-in and attempting a task with a large reward or opting-out and receiving a smaller guaranteed payoff. In the model, I systematically varied: task success probability, variation in success probability, the cost of metacognition and reward associated with task success. Individuals could evolve costly metacognition to estimate their chance of success or evolve a bias to opt-in/out uncritically. Metacognition and a neutral bias evolve under two conditions: firstly, when the environment is uncertain ($\:\mu\:$ = 0.5 and $\:\varphi\:$ close to 1); and secondly (and most strongly) when the task is difficult ($\:\mu\:$ = 0.25) but highly rewarding (*R* > 2) being the only case where maximally costly metacognition evolves. Otherwise, if the variation in task difficulty was low and/or rewards are high, metacognition decreases in favour of a positive bias when tasks are easy or a negative bias when tasks are difficult. Under these conditions, individuals do not benefit from evolving metacognition, and they instead rely on heuristics when making opt-in/out decisions. For example, this could reflect cases where food items are reliably available (Schwartz et al. 2023).

It is somewhat rare for experiments to explicitly consider how ecological factors may predict a species’ metacognition (Beran 2019) and there are very few consistent examples documenting a failure of metacognition (Schwartz 2019). Along with a lack of formal theory, this means it remains unclear when metacognition would be expected to evolve. The taxonomic spread of indicative experimental evidence of metacognition is wide (Perry and Barron 2013; Templer 2019; Tomasello 2023; Watanabe and Clayton 2016). This may imply convergent evolution, as has been suggested for self-recognition (Lage et al. 2022), and/or that the conditions supportive of metacognition are encountered by many different species. As a further clue, rhesus macaques display similar metacognitive illusions to those observed in humans – greater confidence in stimuli that appear easier to remember – suggesting a shared evolutionary origin (Ferrigno et al. 2017).

Of the previous accounts of metacognition, the most widely applicable is the prediction that metacognition should evolve under environmental uncertainty. For instance, when food sources are unpredictable (Schwartz et al. 2023), or when scenarios cannot be responded to by habit (Smith et al. 2012). As an example, metacognition could enable apes to choose between leaping to a branch or using a tool to access out of reach food based on their assessment of their chance of success (Lage et al. 2022). The results of this model support these predictions, given metacognition evolves when task success was most uncertain.

However, this present model also shows that metacognition evolves strongly when the task is difficult and rewarding, even when task success variability is relatively low. This suggests an alternative scenario that favours metacognition beyond that predicted by generalised uncertainty. Here, metacognition is used to identify cases where otherwise difficult to acquire rewards are unusually accessible. One speculative example of this may be omnivore species that can either forage on plants (that may be easier to acquire) or spend time and energy hunting more valuable but elusive prey.

When metacognition evolves in this model, it is usually alongside a neutral bias. The exception is when the task is easy and highly rewarding, where moderate metacognition coevolves with a moderate bias. Under these conditions, selection on increasing metacognition is weak, as mean fitness is roughly equivalent between all values of metacognition. However, the combination of a moderate bias and moderate metacognition may characterise the capuchin. While capuchins do show evidence of metacognition in some scenarios, they frequently fail in metacognitive tasks when compared to other primates (Smith et al. 2018), though this apparent failure is lessened when they are tested on tasks other than the opt-out paradigm (Smith et al. 2020). The reason for this is unclear, though one possibility is their greater risk tolerance (Beran et al. 2016) which may be a consequence of their feeding ecology (Smith et al. 2018). The possibility raised by the results of this model is that the patterns of metacognition observed in capuchins may emerge from repeatedly encountering easy and highly rewarding tasks when the costs of evolving metacognition are low.

It is important to highlight the debates around the use of the opt-out paradigm to study metacognition (Beran 2019; Hampton 2009; Jozefowiez et al. 2009; Smith et al. 2016). Although many studies take extensive steps to eliminate other explanations (Basile et al. 2015), some suggest that no conscious representation of confidence is required to behave optimally in the opt-out paradigm (Hampton 2009). Rather, many instances of seemingly metacognitive behaviour in experiments can be explained through associative learning (Comstock 2019; Hampton 2009). Accordingly, some theorists have proposed a distinction between explicit and implicit metacognition, where the latter involves no conscious representation (Comstock 2019). For this model, I put aside such debates and focused on exploring the conditions under which metacognition would be expected to evolve at all, presupposing that the opt-out paradigm can be a valid measure of metacognition.

The implementation of the opt-out paradigm in this model is very similar to other classes of model such as rational inattention (Maćkowiak et al. 2023) and signal detection models (Sumner and Sumner 2020). In each case, the essence of the problem is the same – the classification of a noisy uncertain signal. Indeed, while this model was framed in terms of a metacognitive problem (estimating success rate), the results would likely be the same if individuals were estimating the reward of the task rather than their chance of success, but this would be less clearly metacognition. As such, this raises the possibility that metacognition is principally a problem of interpreting a noisy signal, where the signal to be estimated is one’s own knowledge or abilities.

There are a few ways this model may be expanded. The model included no social interaction between individuals. This is relevant, as food competition and interactions with conspecifics have been predicted as drivers of social cognition (Schwartz et al. 2023; Tomasello 2023). However, indications of metacognition (deception and sexual mimicry) have been observed in cephalopods that are largely asocial (Schnell et al. 2021), so engaging with conspecifics may not be required for the evolution of metacognition. There are also other benefits to metacognition not considered here such as the rational allocation of limited memory resources (Russek et al. 2024) or greater behavioural flexibility affording generalised foraging benefits or predator avoidance. Additionally, individuals may vary in their ability to succeed after opting-in, for example based on their size or competitive ability. It may be interesting to explore when individuals invest in metacognition versus investing in other traits that merely improve their likelihood of success. A further extension could explore other commonly used experimental designs such as information seeking paradigms (Crystal and Foote 2011) to investigate whether the general predictions from this model hold in different contexts. Previous research has also indicated that capuchins were sensitive to the risk associated with their choice (Beran et al. 2016), suggesting a possible separation between instances of reasoned metacognitive judgements and a reliance on an intrinsic bias. More generally, to better understand the evolution and phylogenetic spread of metacognition, further empirical work is needed to identify conclusive failures of metacognition and to investigate how ecological factors may predict the presence or absence of metacognition.

In summary, I find two conditions that favour the evolution of metacognition if individuals repeatedly encounter opt-out tasks: (1) when the environment is uncertain; (2) when individuals encounter difficult but highly rewarding tasks. Further, selection appeared weak between high and low investment in metacognition when the task was easy and highly rewarding which can result in small investments in both bias and metacognition.

## Data Availability

Model code, data and further figures are available here https://osf.io/x6vwb/?view_only=e5b3467284a44e8d99d8ced033625a96.
